# Recent endeavors on molecular imaging for mapping metals in biology

**DOI:** 10.1007/s41048-020-00118-7

**Published:** 2020-10-31

**Authors:** Jing Gao, Yuncong Chen, Zijian Guo, Weijiang He

**Affiliations:** 1 State Key Laboratory of Coordination Chemistry, School of Chemistry and Chemical Engineering, Nanjing University, Nanjing 210023, China; 2 Chemistry and Biomedicine Innovation Center, Nanjing University, Nanjing 210023, China

**Keywords:** Molecular imaging, Zinc, Copper, Iron

## Abstract

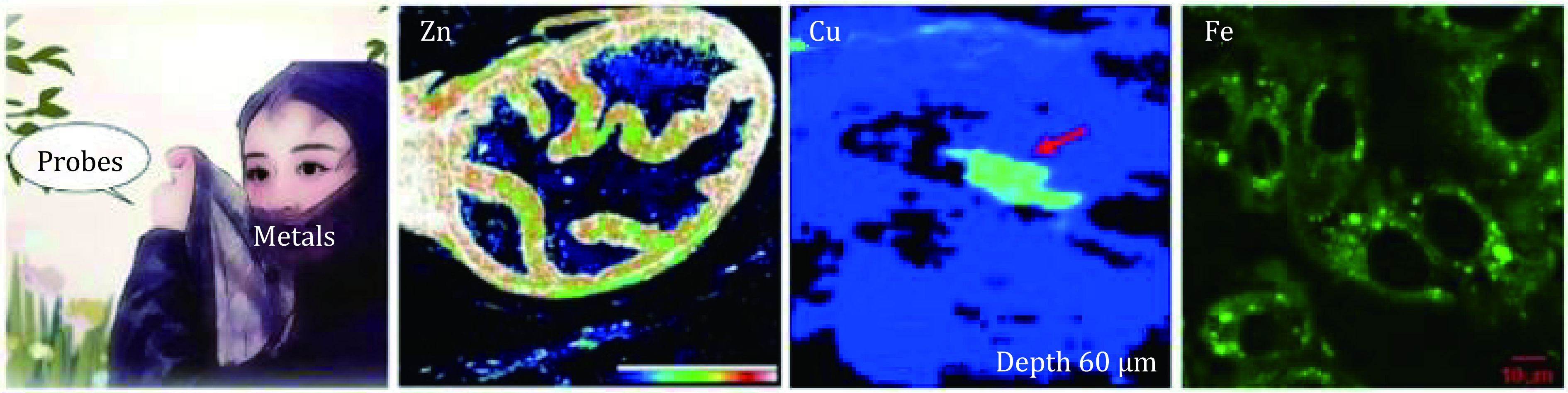

Transition metals such as zinc, copper and iron play vital roles in maintaining physiological functions and homeostasis of living systems. Molecular imaging including two-photon imaging (TPI), bioluminescence imaging (BLI) and photoacoustic imaging (PAI), could act as non-invasive toolkits for capturing dynamic events in living cells, tissues and whole animals. Herein, we review the recent progress in the development of molecular probes for essential transition metals and their biological applications. We emphasize the contributions of metallostasis to health and disease, and discuss the future research directions about how to harness the great potential of metal sensors.

## INTRODUCTION

Iron, zinc, and copper, the top three abundant transition metals in the human body, were well-recognized for their essential roles in maintaining homeostasis and sustaining life. There were numerous examples of clinical diseases that were closely associated with metal misregulation, such as Alzheimer’s disease (AD), amyotrophic lateral sclerosis (ALS) and Parkinson’s disease (PD) (Bleackley and MacGillivray 2011). In this regard, careful maintenance of transition metal homeostasis is required in most living systems.

Labile metal ions, part of the total metal pools, are weakly bound to ligands and can rapidly dissociate (Aron *et al*. 2015). Redox-active labile metals, such as copper and iron, can be harnessed to create new diagnostics and therapeutics (Chang 2015). Ferrous iron (Fe^2+^) is oxidized into ferric iron (Fe^3+^) through reaction with hydrogen peroxide (H_2_O_2_), leading to the formation of highly reactive hydroxyl radicals (OH^·^), referred to as the Fenton reaction. Fe^3+^ is reduced back to Fe^2+^ via reaction with superoxide radicals (O_2_^·–^). This redox cycle is called Harber–Weiss reaction (Hassannia *et al*. 2019). Like iron, copper (Cu^+^ and Cu^2+^) can transform between two oxidation states under biological conditions. These metals can also undergo kinetically appreciable ligand exchange with sensors that respond to metal binding or reaction with an alteration in signal (light or sound) output (Ackerman *et al*. 2017).

Fluorescence imaging (FLI) has been widely used in identifying diversified living processes. Providing a potentially non-invasive set of tools with spatial and temporal resolution, molecular imaging drives scientists to peer into biological environments, and unravels the mysteries of metal ions in a sophisticated milieu. Small-molecule indicators have been assorted into two basic categories for achieving specificity: (1) chelation-based and (2) reaction-based (Ackerman *et al*. 2017). Fundamental mechanisms such as fluorescence resonance energy transfer (FRET), photoinduced electron transfer (PET), and intramolecular charge transfer (ICT) integrate the foundation for most sensor designs (Chang *et al*. 2020). Traditional FLI normally utilizes excitation light < 600 nm, which can lead to auto-fluorescence as well as cell damage, and can be vigorously scattered in deep tissues. To circumvent the high background and low sensitivity, many efforts have been made to develop probes under near infrared (NIR) light excitation and emission. Accordingly, other imaging modalities such as two-photon imaging (TPI), bioluminescence imaging (BLI) and photoacoustic imaging (PAI) have been developed and attracted much attention. Table 1 points to a contrastive analysis of the three imaging modalities.

**Table 1:** Strengths and weaknesses of representative imaging modalities (Baker 2010)

Modality	Labels	Readout	Depth	Resolution	Pros	Cons
TPI	Fluorophores with two-photon absorption (700–1100 nm) cross-section in GM units	Vis–NIR light	~1 mm	1–2 μm	Less photo-damage and photo-bleaching than the corresponding FLI	Relatively poor depth penetration
BLI	Bioluminescent enzymes (typically luciferases)	Light (500– 630 nm emission)	~3 cm	3–5 μm	No external light, zero background emission	Inherently weak emission, expensive (~3 × 10^5^ USD) charge-coupled device (CCD) camera
PAI	Probes (ε > 10 ^4^/(M·cm)) that absorb light (680–950 nm) and create sound signals	Sound	5–7 cm	~50 μm	Better depth than light	Information processing and machines still being optimized

Compared with FLI, BLI does not need an excitation light source, resulting in minimized autofluorescence and much higher signal to noise ratio (Li *et al*. 2013). Beetle luciferases oxidize D-luciferin with ATP and O_2_, generating primarily yellow–green light, whereas marine luciferases release blue photons via the oxidation of imidazopyrazinone analogs (Yao *et al*. 2018). A growing field of “PAI”, which combines optical and ultrasound imaging, shows advantages of high resolution and high contrast at centimeter imaging depths. Theoretically, the design of molecular PAI sensors is primarily based on extending a large π-conjugated backbone with strong PA signal, for example, porphyrin, boron-dipyrromethene (BODIPY), and cyanine derivatives. Excellent reviews of Chan and coworkers discussing the development of PA agents can be found elsewhere (Knox and Chan 2018; Reinhardt and Chan 2018), and fluorescent sensors for measuring metal ions in living systems are also recommended (Carter *et al*. 2014; Liu *et al*. 2013; Park *et al*. 2020; Trusso Sfrazzetto *et al*. 2016).

## METALLOSTASIS

### Zinc

In the cellular environment, zinc is redox inert and is present in the +2 oxidation state. As the second-most abundant metals in the human body following iron, Zn^2+^ is a major cofactor in DNA replication, protein synthesis and cell differentiation (Szewczyk 2013). Some evidence demonstrates that zinc homeostasis in mammals is controlled by the two major families denoted as ZIP (SLC39A) (Eide 2004) and CDF/ZnT (SLC30A) (Palmiter and Huang 2004).

**Figure 1:**
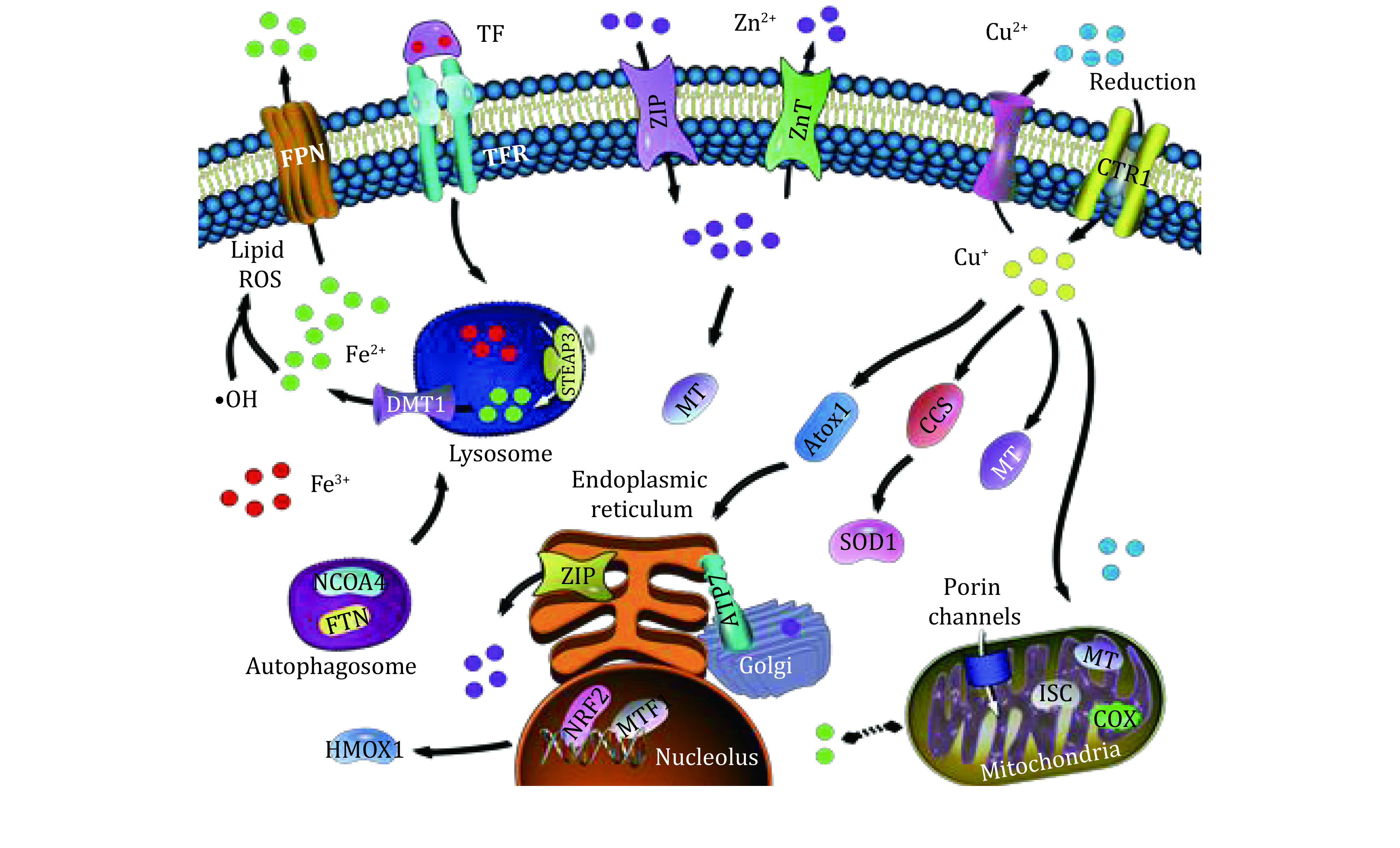
A simplified overview of zinc, copper and iron handling pathways in mammalian cells. ZIP: Zrt/Irt-like protein; ZnT: zinc transport protein; CTR1: copper transporter 1; ATOX1: anti-oxidant 1; ATP7: adenosine triphosphatase 7; CCS: copper chaperones for superoxide dismutase; SOD1: superoxide dismutase 1; MTF1: metal regulatory transcription factor 1; COX: cytochrome c oxidase; MT: metallothionein; ISC: iron-sulfur cluster; TF: transferrin; TFR: transferrin receptor; FPN: ferroportin; FTN: ferritin; NCOA4: nuclear receptor coactivator 4; STEAP3: six-transmembrane epithelial antigen of prostate 3; DMT1: divalent metal transporter 1; NRF2: nuclear factor erythroid 2-related factor 2; HMOX1: heme oxygenase 1

ZIP transporters confer cytoplasmic Zn^2+^ uptake (Huang and Tepaamorndech 2013). Zinc is first absorbed from the diet by ZIP4 (Fig.1), and is delivered to the basolateral membrane or bound to metallothioneins (MTs); the molecular mechanism behind this process has not yet been elucidated (Lazarczyk and Favre 2008; Nishito and Kambe 2018). An excess of unbound cytoplasmic Zn^2+^ is exported outside cells by ZnTs, predominantly ZnT1, and transported to peripheral tissues. Zn^2+^ possibly crosses the outer membrane of mitochondria through porin channels, and then bound to MTs in the intermembrane space or further shipped into the mitochondrial matrix via unknown proteins (Lazarczyk and Favre 2008; Nishito and Kambe 2018). Furthermore, a mutual exchange of Zn^2+^ between the ER and Golgi apparatus through antero- and retrograde vesicular transport has been postulated. Zn^2+^ is delivered in the opposite direction, from organelles to the cytosol by ZIPs, ZIP1 and ZIP7 (ER and Golgi apparatus) are closely related to this process. The ZIP and ZnT families are highly conserved, and their corresponding genes were found in zebrafish as well. The mRNA level of zinc exporter ZnT1 was upregulated in fish subjected to zinc overload and downregulated through zinc deprivation (Zheng *et al*. 2008).

### Copper

As the third-most abundant trace metal in the human body, copper not only plays a critical role in physiological processes (Valko *et al*. 2016), but also associates with cell proliferation and major diseases (Brady *et al*. 2014). It is revealed that biological functions of copper are essentially dependent on Cu^2+^/Cu^+^ transition, also associated with the production of reactive oxidative species (ROS). Abnormal Cu^+^/Cu^2+^ equilibrium can lead to dyshomeostasis, causing many genetic disorders such as Menkes (MNK) disease (Andersson *et al*. 2014), Wilson disease (WD) (Bandmann *et al*. 2015), neurodegenerative diseases (Müller *et al*. 2017), and cancers (Wang *et al*. 2015).

Cu^2+^ is reduced to Cu^+^ by an elusive mechanism and can enter cells through copper transporter 1 (CTR1), the principal high-affinity copper importer (Cotruvo *et al*. 2015). Upon entry, copper interacts with cellular ligands and chaperones that adjust its shipping to specific proteins. Glutathione (GSH) can buffer Cu^+^ pools and mediate Cu^+^ transfer between CTR1 and metallochaperones. Carriage of copper to cytosolic superoxide dismutase 1 (SOD1) proceeds through copper chaperones for superoxide dismutase (CCS). This metallochaperone for Cu/Zn SOD is a known marker for alterations in copper metabolism that inversely correlates with intracellular copper bioavailability (Bertinato *et al*. 2003; Prohaska *et al*. 2003). Metal regulatory transcription factor 1 (MTF1) transcriptionally activates MT genes and other targets. Cytosolic copper is delivered to mitochondria for loading onto cytochrome C oxidase (COX) (Festa and Thiele 2011). Atox1, homolog of Atx1, is responsible for copper delivery to two P-type ATPases, who also participate in copper efflux from cells. They are ATP7A and ATP7B in the trans-Golgi network, where most cuproproteins are metallated. MNK is an X-linked recessive disorder caused by mutations in genes coding for the copper-transport protein ATP7A, leading to copper deficiency. Conversely, WD is a rare genetic disorder characterized by excess copper stored in various body tissues (Bansagi *et al*. 2016).

### Iron

As the most abundant transition metal in the human body, iron is essential and required for a variety of physiological and pathological processes (Theil and Goss 2009). Iron deficiency anemia (IDA) is imposed by a lack of iron, whereas hereditary hemochromatosis (HH) is an iron-overload disorder (Andrews 2000). Once iron is put into circulation, it is first bound to transferrin (TF) as Fe^3+^ and can subsequently enter cells, which maintain iron homeostasis through a balance of import proteins including transferrin receptor (TFR), divalent metal transporter 1 (DMT1), storage proteins like ferritin (FTN), and export proteins like ferroportin (FPN) (Aron *et al*. 2018). It is worth noting that metalloreductase STEAP3 (six-transmembrane epithelial antigen of prostate 3) converts Fe^3+^ to Fe^2+^. In mitochondria, iron-sulfur (Fe/S) cluster proteins (ISCs) are generated from iron and cysteine and are indispensable cofactors for proteins in mitochondrial respiration and other cellular activities (Braymer and Lill 2017).

Aberrant iron levels are implicated in ailments such as pyroptosis and ferroptosis (Zhou *et al*. 2018). Ferroptosis is a newly recognized cell death modality marked by the oxidative modification of phospholipid membranes via an iron-dependent mechanism and that has drawn constantly increasing attention in chemical biology (Conrad *et al*. 2016; Friedmann Angeli *et al*. 2019). Augmented levels of nuclear factor erythroid 2-related factor 2 (NRF2) protein after treatment with erastin and sorafenib (inducers of ferroptosis) render cells resistant to ferroptosis. This protection is traced to upregulation of NRF2 target genes like heme oxygenase 1 (HMOX1). Lysosomes can accumulate a large quantity of iron through degradation of FTN (coined ferritinophagy). Inhibition of lysosomal activity or silencing of nuclear receptor coactivator 4 (NCOA4), a cargo receptor recruiting FTN to autophagosomes for lysosomal degradation and iron release, suppresses ferroptosis (Hassannia *et al*. 2019). Remarkably, a novel quinolone-derived fluorescent turn-on probe IQ44 has been enabling the visualization of autophagosome-lysosome fusion in the process of autophagy (Lim *et al*. 2019).

## MOLECULAR IMAGING

### Zinc

**Figure 2:**
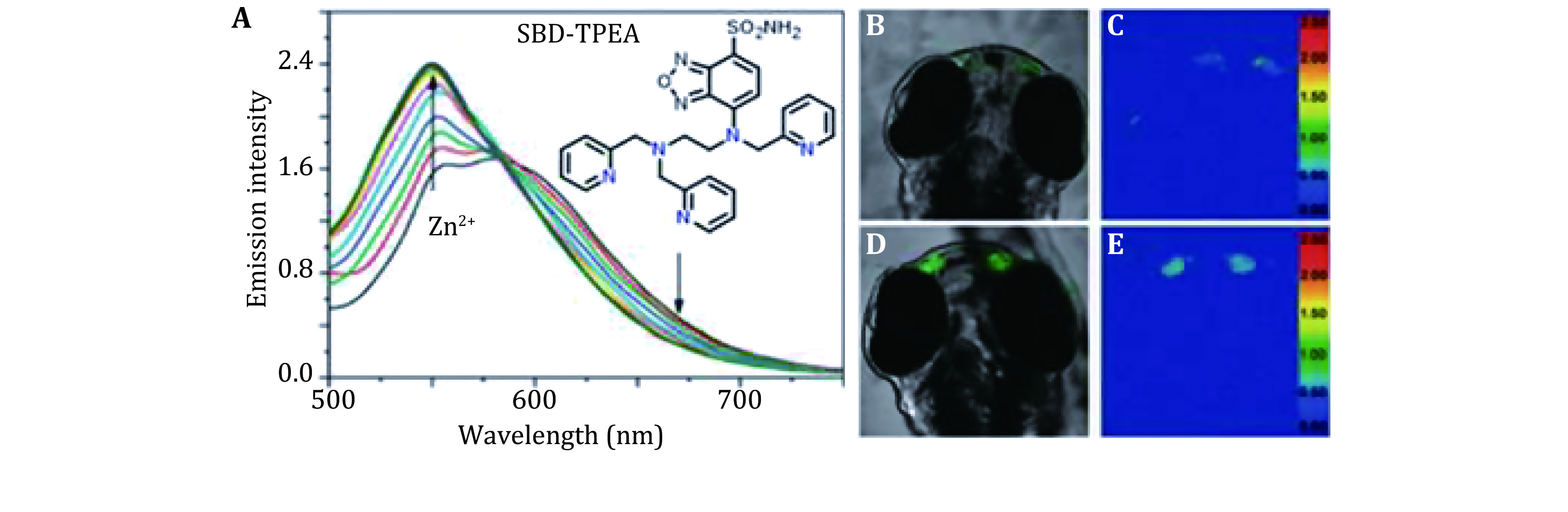
**A** Emission spectra of 3 mmol/L SBD-TPEA (*λ*_ex_ = 460 nm) in buffer (50 mmol/L HEPES/DMSO = 99.85/0.15, pH 7.2) obtained by adding aliquots of Zn^2+^ solution (1.2 mmol/L); Inset in **A**: chemical structure of SBD-TPEA. **B**–**E** Confocal fluorescence ratiometric Zn^2+^ imaging in the head of a 3-day-old zebrafish larva stained with SBD-TPEA. **B** Overlay of bright field and fluorescence image of the SBD-TPEA-stained zebrafish larva from the green channel. **C** Ratiometric image based on the fluorescence images of the zebrafish larva in **B**. **D** Overlay of bright field and fluorescence image of the SBD-TPEA-stained zebrafish larva from the green channel after feeding the larva with Zn^2+^ solution (100 mmol/L) for 1 h. **E** Ratiometric image of the larva in **D**. Green channel images obtained with a bandpath of 510–560 nm, and red channel images obtained with a bandpath of 580–630 nm upon excitation at 488 nm. Adapted from Liu *et al*. 2014

Our laboratory reported a turn-on Zn^2+^ sensor NBD-TPEA, the first example for monitoring zinc ions in neuromasts of zebrafish via fluorescence (Qian *et al*. 2009). By replacing −NO_2_ moiety with −SO_2_NH_2_, we devised an ICT ratiometric fluorescent probe, SBD-TPEA, for Zn^2+^ imaging in biological systems (Liu *et al*. 2014). In the presence of Zn^2+^ (50 mmol/L HEPES/DMSO = 99.85/0.15, pH 7.2), SBD-TPEA exhibits an emission band centered at 585 nm, with an excitation maximum at 466 nm. However, addition of Zn^2+^ to the probe leads to a distinct hypsochromic emission shift from 585 to 545 nm with a well-defined isoemission point at 585 nm. This change is attributed to ICT alteration when Zn^2+^ binds to the probe. The dissociation constant (*K*_d_) of the SBD-TPEA/Zn^2+^ complex was estimated to be 2.1 nmol/L and its limit of detection (LOD) for Zn^2+^ was 0.5 nmol/L. Because SBD-TPEA responded selectively to Zn^2+^ without interference from other metal ions, it was utilized to image Zn^2+^ in HepG2 cells and zebrafish larvae (Fig. 2).

**Figure 3:**
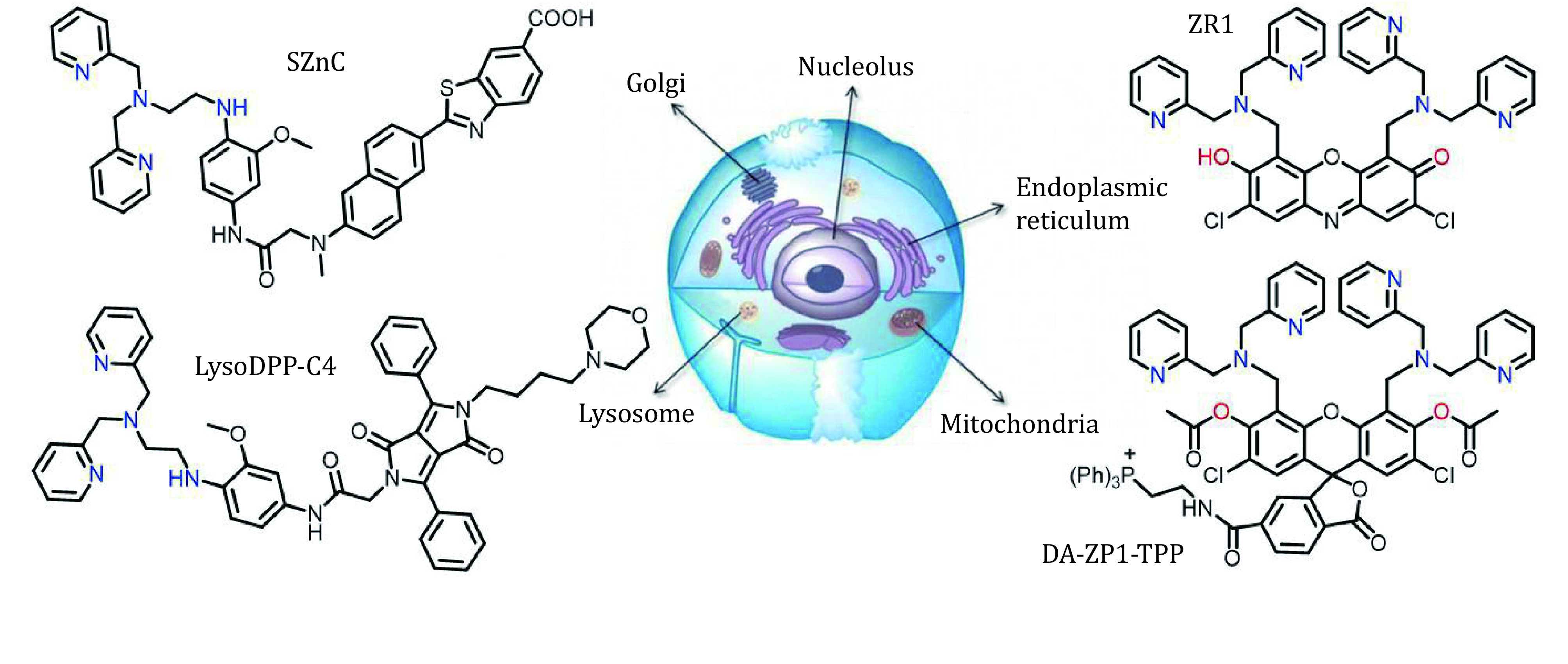
Organelle-specific reversible Zn^2+^ probes. Golgi-targeting SZnC, ER-targeting ZR1, mitochondria-targeting DA-ZP1-TPP, and lysosome-targeting LysoDPP-C4. Adapted from Zhu *et al*. 2016

Through integrating a Zn^2+^ ionophore N,N’-bis(pyridin-2-ylmethyl)ethane-1,2-diamine (BPEA) as the ICT donor of the ANaph fluorophore, we constructed Naph-BPEA with nuclear envelope penetrability (Zhang *et al*. 2013). Of note, this probe did not merely localize in the cell nucleus, it also fluoresced in the cytoplasm. More importantly, our group summarized photoluminescence Zn^2+^ imaging in living subjects before 2015 (Chen *et al*. 2015). Apart from biological functions and sensing mechanisms of labile Zn^2+^, Zn^2+^ roles within an array of subcellular compartments such as mitochondria, lysosome, endoplasmic reticulum (ER), and Golgi apparatus were illuminated in detail, with corresponding Zn^2+^ sensors displayed. Localization is usually defined by comparing the colocalization of the sensor with a well-established organelle marker and quantifying the overlap using Pearson’s correlation coefficient (PCC) given by a imaging software (Carter *et al*. 2014). Organelle-specific Zn^2+^ probes have been developed to study Zn^2+^ distribution in different subcellular compartments (Fig. 3), we will only introduce some representative works due to limited space of this review, readers are encouraged to refer to another review for more detailed information (Zhu *et al*. 2016).

#### Golgi-targeting

Golgi apparatus contain various metalloproteases and alkaline phosphatases, whose catalytic activities rely largely on the Zn^2+^ ions. In 2015, the Kim research group reported a Golgi-localized two-photon ratiometric probe for zinc ions (Singh *et al*. 2015). The dipicolylamine moiety within the compound SZnC selectively makes a complex with Zn^2+^ (30 mmol/L MOP/EtOH = 1:1, pH 7.2), with an emission enhancement peaked at 500 nm. The TP action cross-section values (*Φ*_δmax_) of SZnC in the absence and presence of excess Zn^2+^ were determined to be 16 and 92 GM, while the fluorescence quantum yield *Φ* were 0.12 and 0.93, respectively. Featuring low cytotoxicity and high photostability, this probe can be leveraged to monitor Zn^2+^ fluctuations in real time and getting a 3D distribution picture in the Golgi apparatus.

#### ER-targeting

Metal ions, such as Cu^+^ and Zn^2+^, are required for ER functions. Recently, Lippard and coworkers synthesized a new red-emitting fluorescent probe ZR1 to monitor mobile Zn^2+^ in the ER, a benzoresorufin fluorophore functionalized with two 2,2’-dipicolylamine (DPA) arms (Loas *et al*. 2014). The free probe was non-fluorescent (50 mmol/L PIPES, pH 7.0) due to PET from the Zn^2+^ chelators, while Zn^2+^-binding strongly elevated its emission maximum at 611 nm and an 18-nm Stokes shift. On average, the associated brightness values (*εΦ*) enhanced 44-fold to 2.43 × 10^4^ L/(mol·cm). In comparison with ZBR4, which anchors to the mitochondria, ZR1 spontaneously localizes to the endoplasmic reticulum of HeLa cells.

#### Mitochondria-targeting

Using fluorescein fluorophore instead, Lippard and coworkers presented a reaction-based fluorescent sensor DA-ZP1-TPP, which selectively localized at the mitochondria (Chyan *et al*. 2014). Coordination of Zn^2+^ enhances fluorescence intensity by promoting ester hydrolysis and alleviating PET origination from the DPA motif. Adding nanomolar concentrations of free zinc ions resulted in large increases in both the absorption (*λ*_abs_ = 510 nm) and fluorescence (*λ*_em_ = 529 nm) spectral bands of DA-ZP1-TPP (50 mmol/L PIPES, pH 7.0). These optical changes combined to yield a >140-fold increase in the fluorescence signal ( *Φ*_Zn_ = 0.75). With this probe, it is concluded that tumorigenic cancer cells lose the ability to accumulate Zn^2+^ within their mitochondria in contrast to healthy epithelial prostate cells.

Indeed, close correlations between prostatic Zn^2+^ levels and prostate cancer (PCa) have been reported (Costello *et al*. 2004). At least three ZIPs and six ZnTs are expressed in a lobe-dependent manner in the prostate (Kelleher *et al*. 2011). In malignant prostate tissues, there is a dramatic decline in Zn^2+^ concentration (500 nmol/g vs. 3000 nmol/g for cancerous and healthy tissues, respectively), as well as a downregulation of ZIP1 (Kolenko *et al*. 2013).

#### Lysosome-targeting

Very recently, LysoDPP-C4 has been developed for the evaluation of low pH and Zn^2+^ in an AND logic fashion to investigate lysosomal Zn^2+^ in prostate cancer cells (Du *et al*. 2019). A morpholine unit is appended for targeting lysosomes, as confirmed by HeLa cell fluorescence imaging studies. LysoDPP-C4 demonstrated an excellent selectivity toward Zn^2+^ and the resulting LysoDPP-C4/Zn^2+^ complexes proved insensitive to pH 3.5–9. At low pH, Zn^2+^ chelation contributes to a large increase in the fluorescence intensity at 515 nm (*λ*_ex_ = 430 nm) ascribed to suppression of PET. The sensor formed a host-guest complex in 1:1 stoichiometry with *K*_d_ = 1.91 nmol/L. Besides, histological studies using a human sample revealed that LysoDPP-C4 could distinguish between cancerous prostate tissue and healthy one.

In view of the advances in Zn^2+^-specific chelators (TPEA, BPEA, DPA, *etc*.) and Zn^2+^-complexation-induced spirolactam ring-opening or hydrolysis (Jin *et al*. 2018), concurrent with phosphorescence lifetime imaging (PLIM) of labile Zn^2+^ (Zhang *et al*. 2018), it is not surprising that Zn^2+^ sensors constitute the largest family of fluorescent indicators for transition metals. Some of them are reversible and ratiometric, applied to appealing models. So far, the physiological participation of this ion is not completely clear in spite of the large arsenal of fluorescent Zn^2+^ sensors.

### Copper

Molecular sensors are intriguing tools to visualize the distribution and speciation of labile copper. Nevertheless, there are added challenges posed by aiming at labile copper over Zn^2+^ due to the need for discrimination between different oxidation states, the quenching nature of Cu^2+^, and the fact that probes must have enough affinities to compete for copper within its biological window (10^−21^−10^−17^ M) (Wegner *et al*. 2011). In consequence, only a fraction of copper sensors has been utilized for biologically accessible copper. The Kim group shed light on the pathophysiological part of copper ions and sensors for the measurement thereof (Peter *et al*. 2015).

#### Cu^+^

**Figure 4:**
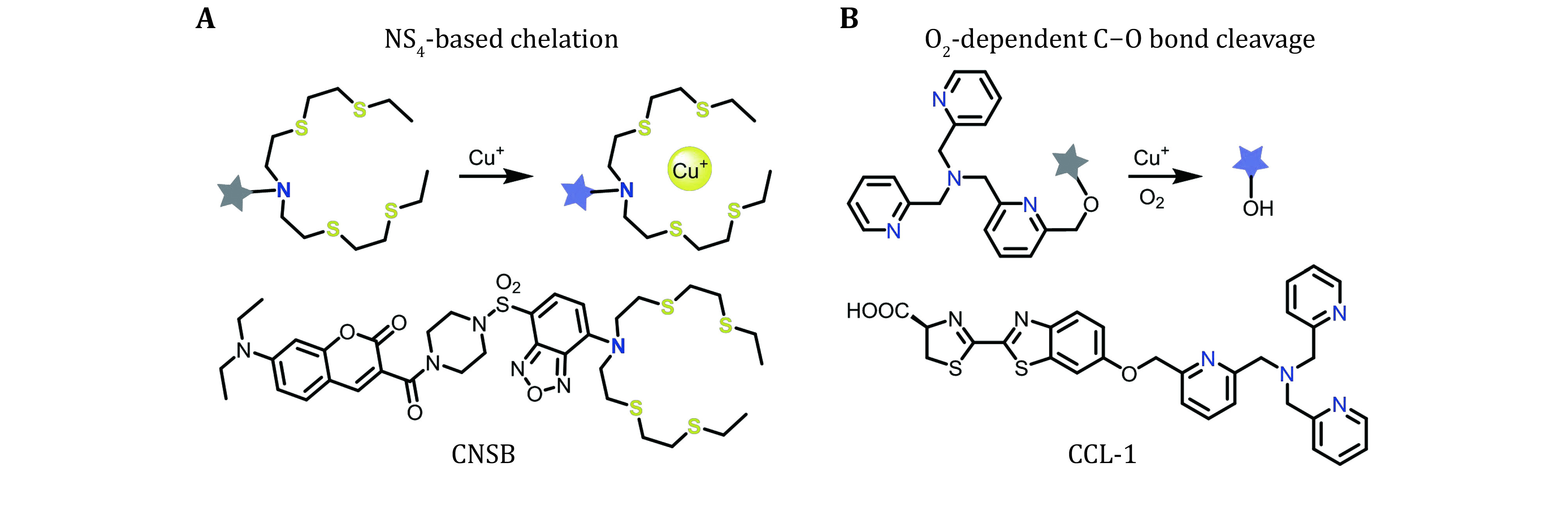
Design approaches and selected structures for Cu^+^ detection. **A** NS_4_-based chelation: CNSB. **B** O_2_-dependent C−O bond cleavage: CCL-1

Although Cu^+^ is prone to disproportionation and acquires stabilization by specific ligands in aqueous media (Paredes and Das 2011), it is considered to be the dominant intracellular copper oxidation state of labile copper pools (Festa and Thiele 2011). Most of the probes designed for biological systems target Cu^+^, engaged in two common aspects as follows (Fig. 4).

(A) NS_4_-based chelation

Thioether (NS_4_) receptors can be modified to develop fluorescent Cu^+^ sensors. One notable example is CTAP-1, the first small-molecule sensor for mapping labile Cu^+^ in cells (Yang *et al*. 2005). Soon after this study, the Chang group presented coppersensor 1 (CS1) with visible excitation and emission profiles (Zeng *et al*. 2006). Tuning the BODIPY fluorophore of CS1 to a rhodol scaffold produced a more hydrophilic Cu^+^ sensor called copper Fluor-3 (CF3) (Dodani *et al*. 2014). As described earlier, fluorescent sensors that excited at short wavelengths succumb to limited penetration depths, high autofluorescence, and inevitably induce photobleaching and photodamage. These flaws hinder their use for long-term imaging in tissues and organisms. One way to remove these limitations is by NIR single-photon excitation (Guo *et al*. 2014). Cao Cu-3 represents a novel NIR fluorescent turn-on probe suitable for imaging endogenous Cu^+^ ions in living systems (Cao *et al*. 2012).

Another approach is to use two-photon microscopy (TPM), employing NIR photons of lower energy as the excitation source to possess the advantage of deepened penetration (>500 μm) (Kim*et al*. 2014). Our laboratory presented the first-generation ER-localized TP Cu^+^ sensor CNSB (Guo *et al*. 2019). There is a distinct overlap between coumarin-A (donor) emission and 4-amino-7-sulfamoyl benzoxadiazole (ASBD, acceptor) absorption, implying these two compounds are qualified candidates as a FRET pair to compose ratiometric sensors. CNSB exhibits ~7.44 × 10^−11^ M *K*_d_ value (10 mmol/L Tris-HCl/DMF = 6:4, pH 7.2) and the Cu^+^-enhanced emission ratio F_470_/F_565_ is stable from pH 4.0 to 8.0. The sensor was applied to assessing Cu^+^ fluctuation in MCF-7 cells pre-incubated with tunicamycin, proving the relationship between Cu^+^ augmentation and ER stress. Meanwhile, the spatial distribution of Cu^+^ in the heart slice of a 2-week rat was observed via TPI owing to the two-photon ability of coumarin fluorophore. Negligible enhancement of coumarin emission was observed during the fluorescent titration of CNSB.

(B) O_2_-dependent C−O bond cleavage

**Figure 5:**
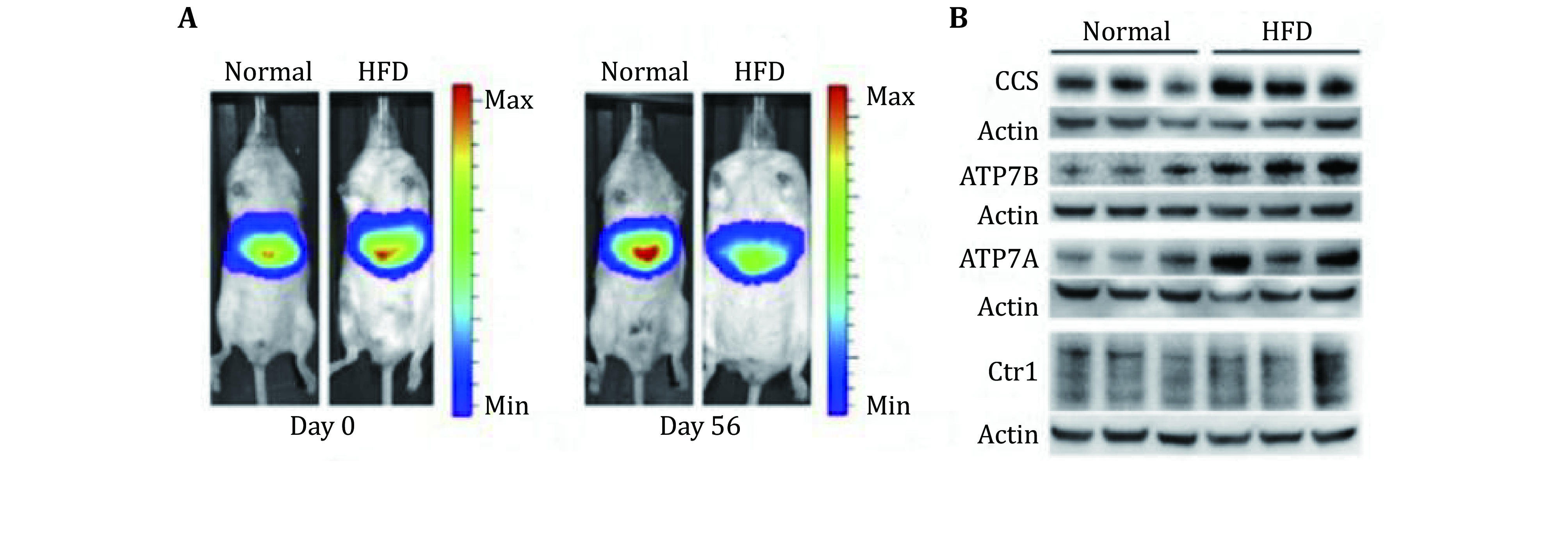
CCL-1 imaging reveals hepatic copper deficiency in a diet-induced murine model of NAFLD. **A** Representative images of mice from HFD and normal diet groups before the study (day 0) and at the end of the 8-week feeding period (day 56). **B** Western blot analysis of liver extracts of three mice fed normal diets and three HFD mice. Adapted from Heffern *et al*. (2016)

Inspired by the O_2_ activation for copper-dependent enzymes, the Chang group adapted the Cu^+^ chelator tris(2-pyridylmethyl)amine (TPA) to develop copper-caged luciferin-1 (CCL-1) (Heffern *et al*. 2016), a bioluminescent reporter for monitoring labile Cu^+^ levels in a diet-induced mouse model of non-alcoholic fatty liver disease (NAFLD) that manifests as a hepatic copper deficiency, revealing altered expression levels of central copper trafficking proteins that accompany symptoms of glucose intolerance and weight gain (Fig. 5). As supported by Western blot, CCS levels were elevated in the high-fat diet (HFD) mice over control diet mice, and the major copper exporter proteins ATP7A and ATP7B were also up-regulated.

Not long ago, linking fluorescein donor and rhodamine acceptor through the TPA bridge, they reported reaction-based ratiometric FRET copper probe FCP-1. Together with its 2-color response (F_526_/F_576_), collective results indicate that oncogene-driven changes in the metabolism of GSH, a major cellular redox buffer, leads to a labile Cu^+^ deficiency with differential expression of CTR1 (Chung *et al*. 2019). This work connects Cu^+^ dysregulation and GSH stress in cancer, providing a roadmap for studying the crosstalk between metal and redox pathways in physiology and pathology with probes.

#### Cu^2+^

**Figure 6:**
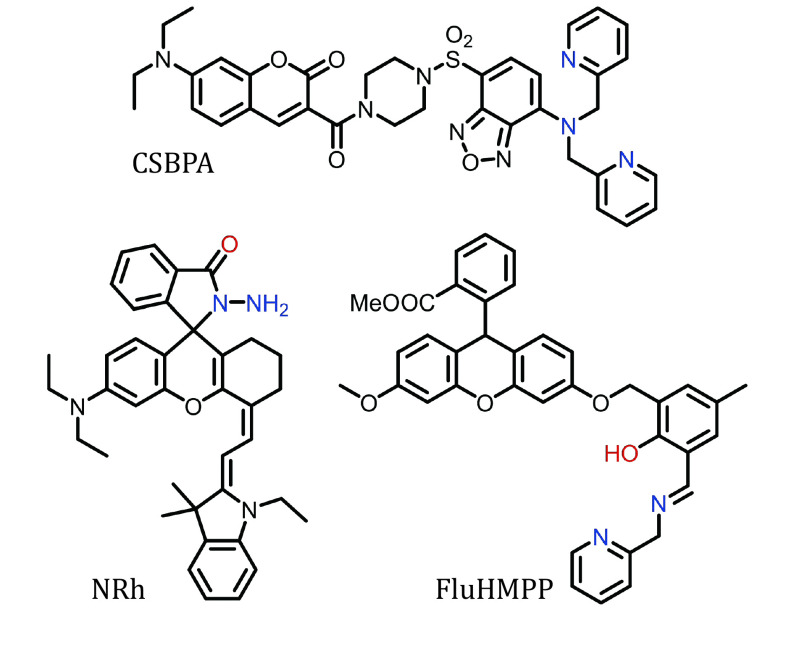
Selected structures of Cu^2+^-responsive probes based on chelation (CSBPA), hydrolysis (NRh) and oxidation (FluHMPP)

As synthetic fluorescent probes for monovalent copper have been thoroughly reviewed by others (Cotruvo *et al*. 2015; Fahrni 2013), we focus our discussion on Cu^2+^ probes that compensate for these areas of copper sensing. A large library of luminescent chemodosimeters for Cu^2+^ bioimaging has been collected by Fuyou Li *et al*. (Yang *et al*. 2013). Most of such probes recognize Cu^2+^ by three principal categories: chelation, hydrolysis and oxidation (Fig. 6). Among them, the oxidation pattern draws in the fewest probes while the other two seem more attractive.

(A) Chelation

As mentioned previously, 2,2’-dipicolylamine (DPA) can be manipulated to design probes selective for Cu^2+^ over Zn^2+^ (Ballesteros *et al*. 2009). Modifying the FRET pair of CNSB with DPA unit forms a reversible ratiometric sensor CSBPA for intracellular Cu^2+^ imaging (Chen *et al*. 2013). Later we achieved *in vivo* fluorescence imaging for Cu^2+^ in live mice for the first time by a BODIPY-derived NIR fluorescent sensor BDPA (Xue *et al*. 2016), profiting from its large Stokes shift (~100 nm), excellent photostability and high quantum yield (*Φ* = 0.85).

**Figure 7:**
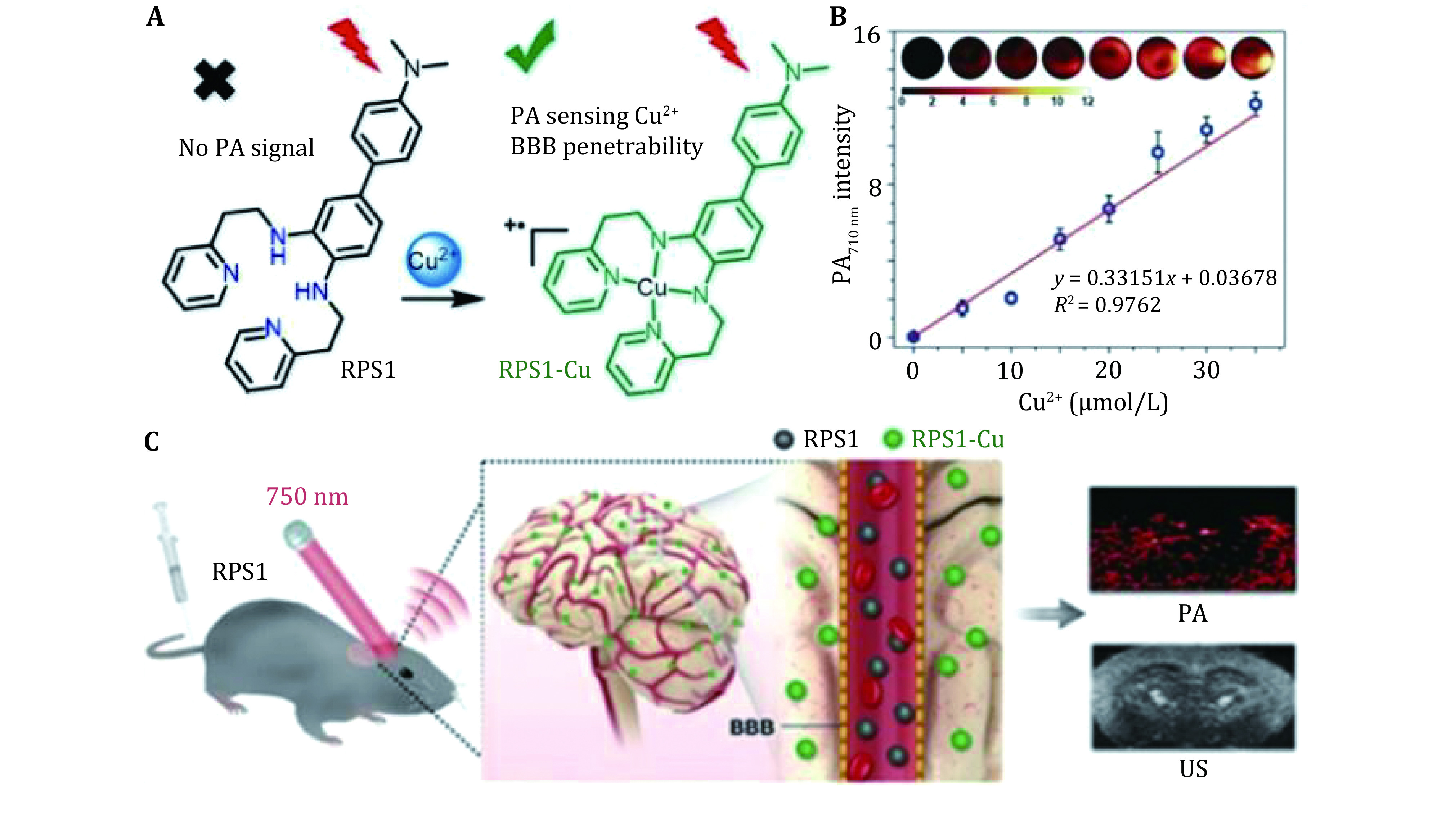
**A** Experimental design of target PA probe RPS1 for Cu^2+^ analysis. **B** Calibration curve of the PA intensity at 710 nm for measuring Cu^2+^ in the range of 0–35 μmol/L. **C** Schematic diagram of RPS1 crossing the BBB and detecting Cu^2+^, as observed by PAI. Adapted from Wang *et al*. 2019a

Wang *et al*. developed a series of activatable PA probes with low molecular weights (<438 Da), and could specifically chelate with Cu^2+^ to form radicals displaying turn-on PA signals in the NIR region (Wang *et al*. 2019a). Introducing the electron-donating group N,N-dimethylaniline into the probe was found to significantly enhance the radical stability and PA intensity. The best probe in the series, RPS1, produced a fast response (within seconds) to Cu^2+^ with a low LOD of 90.9 nmol/L (0.02 mol/L PBS, pH 7.4). Owing to the low molecular weight and amphiphilic structure, RPS1 could effectively cross the blood−brain barrier (BBB) and thus maps Cu^2+^ in the brains of AD mice via PAI for the first time (Fig. 7).

(B) Hydrolysis

The group headed by Chan reported the synthesis and photoacoustic properties of APC-1 and APC-2, two ratiometric probes for mobile Cu^2+^ (Li *et al*. 2015). Upon binding Cu^2+^, the sensors display 89- and 101-fold enhancements of normalized ratiometric turn-on responses, respectively. Both APCs are equipped with a 2-picolinic ester sensing module that is readily hydrolyzed in the presence of Cu^2+^ but not by other divalent metal ions. Additionally, ratiometric PAI was realized by using an aza-BODIPY dye scaffold exhibiting two spectrally resolved NIR absorbance bands, which correspond to the 2-picolinic ester capped and uncapped phenoxide forms.

**Figure 8:**
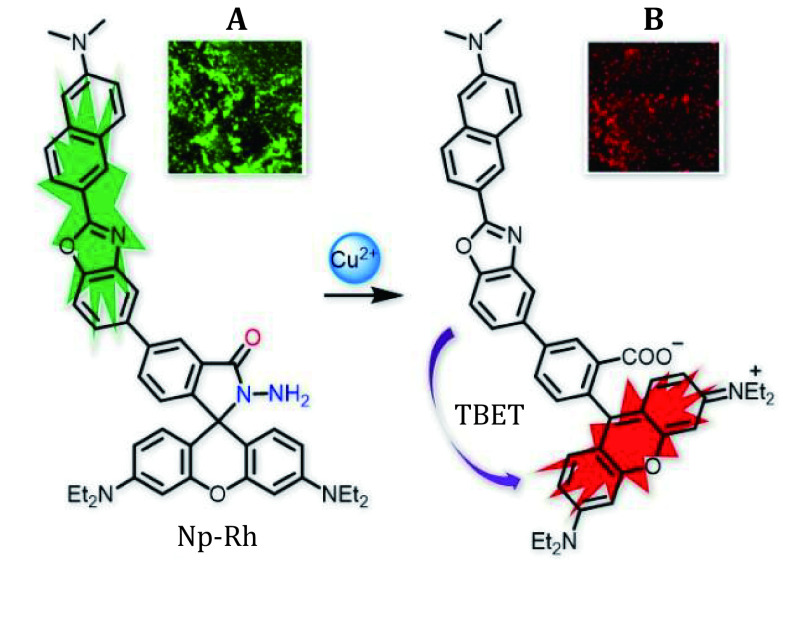
Two-photon TBET probe Np-Rh. TPI of a rat liver frozen slice stained with 10 μmol/L Np-Rh at ~95 μm for 60 min (**A**), followed by treatment with 100 μmol/L Cu^2+^ and incubated for another 60 min (**B**). Green channel images obtained with a bandpath of 450–530 nm (**A**), and red channel images obtained with a bandpath of 540–650 nm upon excitation at 780 nm with femtosecond pulses (**B**). Adapted from Zhou *et al*. 2014

Combining Cu^2+^-promoted hydrolysis with spirolactam ring-opening generates an effective strategy to construct fluorescent Cu^2+^ chemodosimeters. A ratiometric TP probe for Cu^2+^ trafficking based on through-bond energy transfer (TBET), was designed by directly conjugating BODAN (donor, green emission) and rhodamine spirolactam (acceptor, red emission) (Fig. 8) (Zhou *et al*. 2014). Np-Rh elicits a ratiometric response (EtOH/H_2_O = 1:9) upon addition of Cu^2+^, with highly efficient energy transfer (93.7%) and two well-resolved emission peaks separated by 100 nm. Moreover, the Cu^2+^ distribution in Np-Rh-labeled HeLa cells as well as rat liver frozen slices pretreated with Cu^2+^ was visualized using TPM in the 450–530 and 540–650 nm collection windows.

Similarly, Fuyou Li and coworkers introduced a frequency upconversion luminescence (UCL) Cu^2+^ chemodosimeter NRh (Liu *et al*. 2016), lighting up efficient single-photon upconversion emission at 730 nm under NIR excitation at 808 nm. This probe can serve as an ideal Cu^2+^ sensor for *ex vivo* and *in vivo* assay in a WD mouse model. Impressively, the exploitation of UCL small-molecule probes and their bioimaging applications have been attracting much attention (Dong *et al*. 2018; Yang *et al*. 2016; Zhang *et al*. 2017).

(C) Oxidation

**Figure 9:**
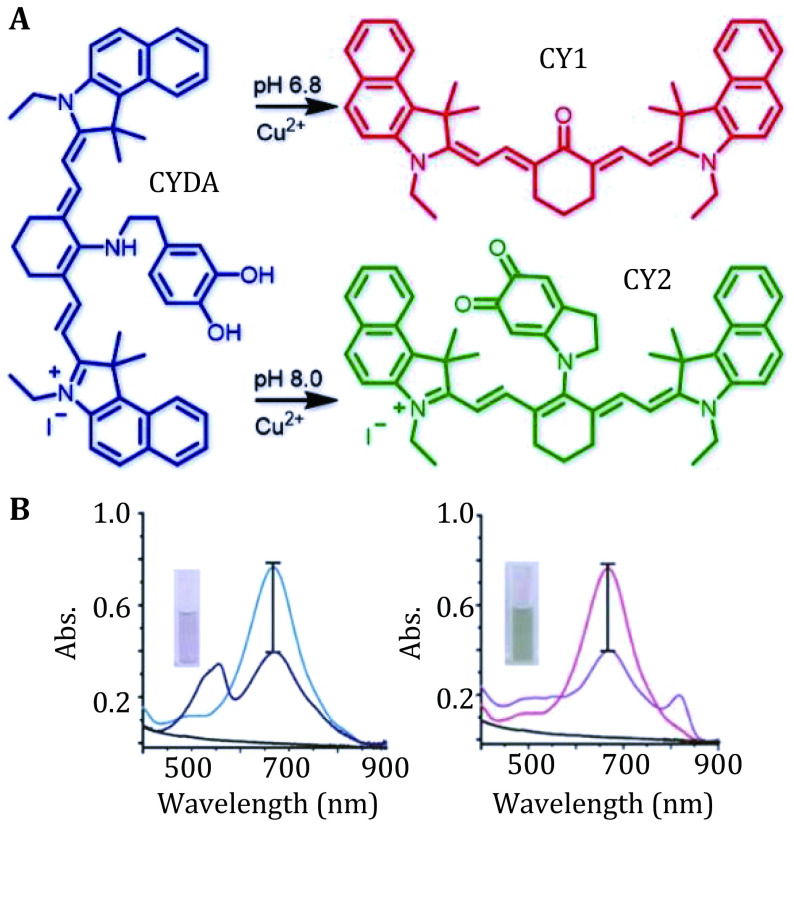
**A** Oxidation of CYDA by Cu^2+^ in acidic solution (pH 6.8) and alkaline solution (pH 8.0) respectively. **B** UV−Vis−NIR absorption spectra (WD patients’ urine/EtOH/HEPES = 10:10:1) of 10 μmol/L CYDA when the pH value was 6.8 (left), 8.0 (right), from top to bottom: CYDA/Urine_start_, CYDA/Urine_end_ and Urine. Adapted from Shi *et al*. 2018

Subsequently, the same group developed an easy-to-use probe CYDA based on UV–Vis–NIR absorption changes with excellent sensitivity and selectivity for Cu^2+^ (Shi *et al*. 2018). The mechanism of CYDA oxidation by Cu^2+^ was first explored (Fig. 9). In aqueous solution (EtOH/H_2_O = 1:1), addition of Cu^2+^ to CYDA induced significant changes in the absorption spectra. When the solution was mildly acidic (pH 6.8), the absorbance at 670 nm went down, with the absorbance at 553 nm (*ε* = 1.09 × 10^5^ L/(mol·cm)) up, accompanied by the solution color changes from blue to red. In alkaline solutions (pH 8.0), the absorbance at 670 nm (*ε* = 2.14 × 10^5^ L/(mol·cm)) dropped gradually, whereas the absorbance at 823 nm (*ε* = 0.22 × 10^5^ L/(mol·cm)) rose with an isosbestic point at *λ* = 793 nm. In the meantime, the solution color turned into greyish blue. These phenomena confirmed the formation of CY1 and CY2. They further demonstrated that the probe was able to quantify Cu^2+^ in urine from WD patients.

Even a bit earlier than the above study, oxidative C−O bond cleavage turn-on probe FluHMPP has been prepared (Shi *et al*. 2013). On addition of 20 equiv of Cu(NO_3_)_2_ (10 mmol/L Tris-HCl/CH_3_CN = 7:3, pH 7.2), there was a strong emission enhancement (30-fold) after 2 h. Confocal microscopy experiments have demonstrated that FluHMPP was membrane permeable and capable of tracing exogenous CuCl_2_ in HeLa cells. Nevertheless, such C=N structures are unstable and sensitive to reactive oxygen species.

### Iron

Despite great interest in labile iron pool (LIP), its biological function remains insufficiently understood, in part due to a relative lack of tools for directly assessing labile iron in living specimens (Dixon and Stockwell 2014). Molecular imaging using iron selective sensors opens up a broad avenue for better understanding the complex handling of iron in biology. Ideally, probes should be able to track biological iron status, selectively detecting either Fe^2+^ or Fe^3+^ or responding to iron species such as heme-iron or ISCs. Because Fe^2+^ and Fe^3+^ are potent emission quenchers, iron imaging within living cells encountered many challenges. Two early sensors, Calcein and Phen Green SK, exhibit turn-off responses to iron binding (Carter *et al*. 2014). However, both probes cannot adequately distinguish the two oxidation states of iron but can interact with other metal ions, leading to an interfering signal. In the past few years, a substantial body of efforts has been devoted toward construction of iron probes that may make a stunning breakthrough regarding cellular iron homeostasis.

#### Fe^2+^

**Figure 10:**
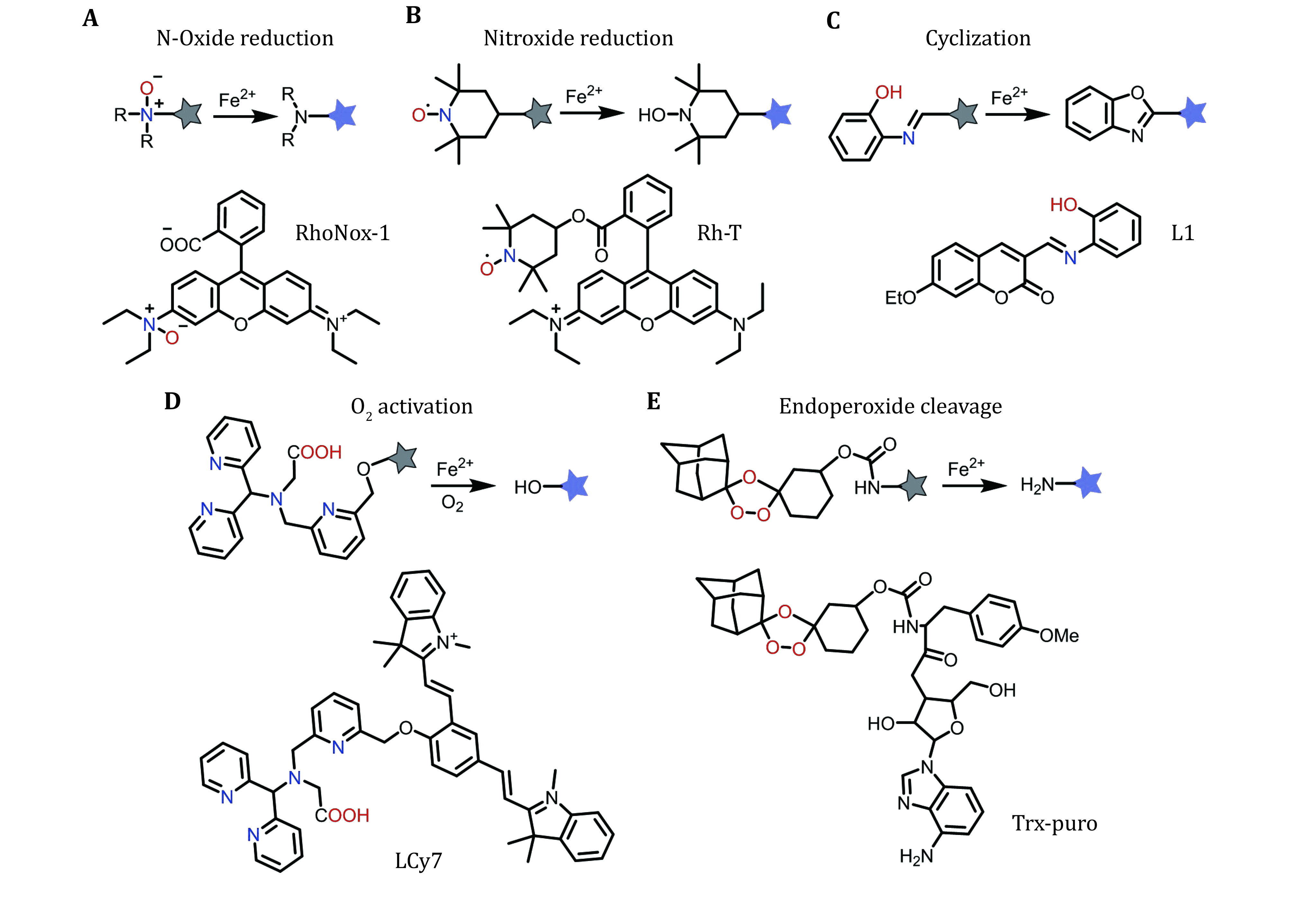
Reaction-based sensing probes for Fe^2+^ detection. **A** N-oxide: RhoNox-1. **B** Nitroxide reduction: Rh-T. **C** Cyclization: L1. **D** O_2_ activation: LCy7. **E** Endoperoxide cleavage: Trx-puro

The growing palette of chemodosimeters for Fe^2+^ mapping exploits the potent redox activity of this metal ion, and the last several years have witnessed a tremendous number of novel Fe^2+^ chemoprobes suitable for live-cell imaging experiments. Recent reviews by Hirayama, Aron and coworkers exhaustively profiled the development of molecular probes for Fe^2+^ (Aron *et al*. 2018; Hirayama 2019). A palette of Fe^2+^-mediated principles have been reported, including N-oxide or nitroxide reduction, cyclization, O_2_ activation and endoperoxide cleavage (Fig. 10).

(A) N-oxide reduction

The deoxygenation of tertiary amine N-oxide is preferably mediated by Fe^2+^ as it has a lower redox state than Fe^3+^. Utilizing the reaction, Hirayama and coworkers developed the first turn-on fluorescent Fe^2+^ probe RhoNox-1 (Hirayama *et al*. 2013), which has been commercially available and applied to detecting Fe^2+^ changes during ferroptosis (Wang *et al*. 2019b). To date, this Fe^2+^ sensing strategy has emerged as the most widely used one, establishing a series of sensors. For instance, HMRhoNox for visualization of intracellular Fe^2+^ delivered by TF (Niwa *et al*. 2014), CoNox-1 and FluNox-1 for scrutiny of intracellular redox equilibrium shift toward Fe^2+^ in hypoxic HepG2 cells (Hirayama *et al*. 2017), and a membrane-anchoring probe Mem-RhoNox to scrutinize Fe^2+^ release during endocytotic iron uptake (Niwa *et al*. 2018).

**Figure 11:**
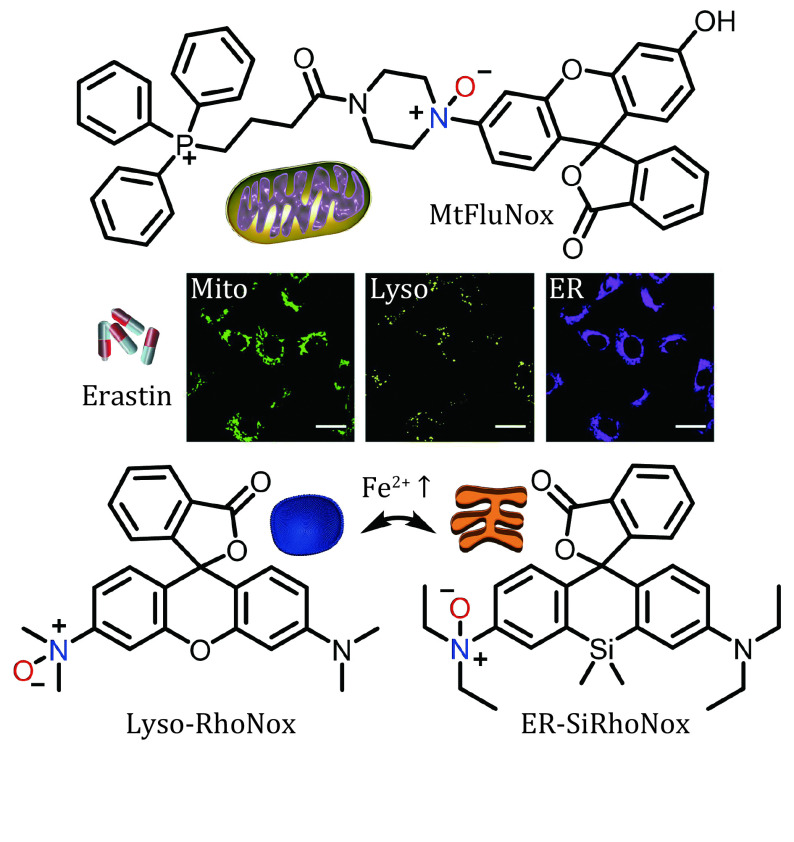
Chemical structures of the fluorescent Fe^2+^ probes used MtFluNox, Lyso-RhoNox and ER-SiRhoNox. And fluorescence microscopic analysis for multi-color detection of Fe^2+^ at each targeted organelle. Adapted from Hirayama *et al*. 2019b

Recent research expanded the applicability to establish a series of organelle-specific fluorescent probes selective to Fe^2+^, *i.e*., MtFluNox, Lyso-RhoNox, ER-SiRhoNox and Gol-SiRhoNox. The former three chemosensors (Fig. 11) demonstrated similar off/on contrast and reaction rates (2.1 × 10^−3^ s^−1^, 2.2 × 10^−3^ s^−1^, and 1.7 × 10^−3^ s^−1^), investigated Fe^2+^ specifically at the targeted organelles (*PCC* = 0.81, 0.80, 0.80), and depicted fluorescence enhancement at 535 nm (green, 100-fold), 575 nm (orange, 60-fold), and 660 nm (magenta, 60-fold), respectively. Furthermore, these probes indicated the aberrant elevation of labile Fe^2+^ levels in the lysosomes and ER but no Fe^2+^ fluctuation in the mitochondria prior to HT1080 cell death initiated by erastin (Hirayama *et al*. 2019b).

As depicts in Fig. 1, DMT1 serves as a primary transporter of Fe^2+^. Over the course of the endocytotic process, directing DMT1 toward the cellular membrane via the trans-Golgi network is partially regulated by a retromer-mediated protein-sorting machinery comprising vacuolar protein-sorting proteins (VPSs) (Burd and Cullen 2014). Therefore, the Golgi organelle functions as a node in this dynamic system for intracellular Fe^2+^ species along with DMT1. Dysfunction of VPSs, particularly the mutation of VPS35, can activate the aberrant delivery of DMT1 to lysosomes concomitantly with Fe^2+^ ions (Tabuchi *et al*. 2010). The last one Gol-SiRhoNox revealed an abnormal cellular Fe^2+^ distribution induced by dysfunction of VPS35 with a molecular chaperone R55 (Hirayama *et al*. 2019a).

(B) Nitroxide reduction

In parallel studies, a related Fe^2+^-promoted nitroxide reduction reaction has been employed for reaction-based sensing of Fe^2+^ (Maiti *et al*. 2015). A rhodamine-linked nitroxide probe, Rh-T, bears a pendant paramagnetic 2,2,6,6-tetramethylpiperidine-1-oxyl (TEMPO) group that quenches fluorescence. Fe^2+^ reduces the receptor radical to a diamagnetic hydroxylamine, resulting in a significant fluorescence turn-on (~2.5-fold) with a LOD ~0.75 µmol/L. Fe^2+^-dependent reduction of the TEMPO radical of Rh-T triggers both a fluorescence turn-on and a change in electron paramagnetic resonance (EPR) signal. Following this example, Zhu group reported NT-Fe with the fastest response (<50 s), as well as the sensitive LOD of 89 nmol/L (Zhang*et al*. 2020). In zebrafish, NT-Fe responds to the addition of exogenous Fe^2+^, and its signal decreases when pretreated with Fe^2+^ scavenger 2,2’-bipyridine (Bpy).

(C) Cyclization

By exploiting the Lewis acidity of Fe^2+^, a recent report highlighted the application of an Fe^2+^-specific cyclization of a phenolic unit adjacent to a C=N bond appended to a coumarin derivative (Long *et al*. 2018). Upon cyclization, generation of a benzoxazole ring handicaps C=N bond isomerization and rotation, turning on fluorescence in aqueous solution and HepG2 cells, which has also been confirmed by TD-DFT calculation. Significantly, the sensing reaction completed in 2 min with high sensitivity (LOD = 45 nmol/L). However, more analogous probes should be rationally constructed to verify the validity of the proposed mechanism.

(D) O_2_ activation

Prior work by Chang laboratory on creating Cu^+^ indicators, presaged the general utility of biomimetic oxygen activation for Fe^2+^ surveillance. Inspired by the O_2_ chemistry of Fe^2+^, and in particular, the 2-His-1-carboxylate triad motif found in mononuclear non-heme Fe^2+^ enzymes (cytochrome P450), they engineered iron probe 1 (IP1) (Au-Yeung *et al*. 2013). IP1 was capable of monitoring elevated intracellular labile Fe^2+^ levels in HepG2/C3A cells caused by treatment with hepcidin or ascorbic acid.

Replacement of the fluorescein alcohol substituent with a cyanine dye (Cy7), the Tang group yielded another NIR fluorescent probe (LCy7) (Wu *et al*. 2020), comprising a tris(pyridine) carboxylate-type ligand cage as an iron recognition and activity site. Upon Fe^2+^ coordination, O_2_ activation triggers intramolecular C–O bond cleavage and oxidation to release the fluorophore, resulting in an increase (~3.7-fold) in the fluorescence emission peaked at 690 nm. Utilizing LCy7, dynamic Fe^2+^ enhancement was distinctly monitored in HL-7702 cells under ER stress by acetaminophen (APAP) stimulation. *In* *vivo* FLI disclosed the conspicuous Fe^2+^ ascent in the liver of mice during drug-induced liver injury (DILI).

(E) Endoperoxide cleavage

Motivated by Fe^2+^-reactive endoperoxide activation observed in antimalarial drugs (*e.g*., Artemisinin), Renslo and coworkers designed Trx-puro, a histochemical stain for labile Fe^2+^ comprised of a trioxalane-caged puromycin (Spangler *et al*. 2016). Upon reaction of the probe with Fe^2+^, free puromycin is released, which can be translated onto nascent peptides and quantified via a conventional immunostaining method by a puromycin antibody after fixation. Application of Trx-puro to a panel of cancer cells (PC-3, U-2 OS, MCF10A and RKO) reveals FTN/FPN overexpression and declined Fe^2+^ stores. Linking fluorescein and Cy3 through this bioinspired endoperoxide trigger, Chang *et al*. envisioned FRET iron probe 1 (FIP-1) for ratiometric fluorescence Fe^2+^ imaging upon 35MEW28-induced ferroptosis in MDA-MB-231 cells (Aron *et al*. 2016).

**Figure 12:**
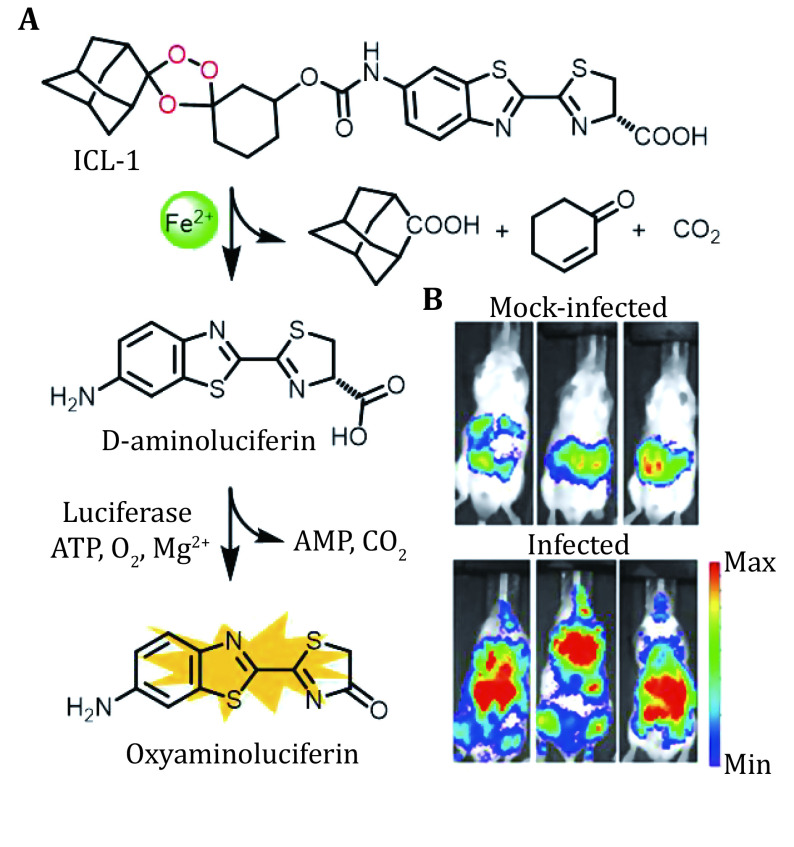
**A** Fe^2+^-selective cleavage of ICL-1, an endoperoxide-luciferin conjugate and *in vivo* probe of Fe^2+^. **B** Representative images of FVB-Luc^+^ mice mock-infected (PBS) or infected with *A. baumannii* through retroorbital injection (dorsal images at 30 min postinjection of ICL-1) and imaged with ICL-1 (25 nmol) at 24 h postinfection. Adapted from Aron *et al*. 2017

Expanding the scope of oxidative cleavage to BLI, the same group then reported the first bioluminescent indicator for Fe^2+^ (Aron *et al*. 2017), iron-caged luciferin-1 (ICL-1), to surveil labile Fe^2+^ accumulation in a luciferase-expressing murine (FVB-Luc^+^) model of *Acinetobacter* (*A*.) *baumannii* infection (Fig. 12). The probe also retains high selectivity and sensitivity for Fe^2+^ over a variety of biologically-relevant metal ions, oxidants, and reductants and is feasible for detecting both Fe^2+^ rise and fall in PC3M-luc cells and other Fe^2+^-supplemented/depleted models.

Despite the exquisite selectivity toward Fe^2+^ for bioimaging, these reaction-based probes were not shown to have a reversible monitoring mode, hampering the capability to probe Fe^2+^ fluxes. If a probe is able to synchronously measure Fe^2+^ and Fe^3+^ without interference from other metal ions, the privileged part of iron taking in ferroptosis will no longer remain a mystery.

#### Fe^3+^

**Figure 13:**
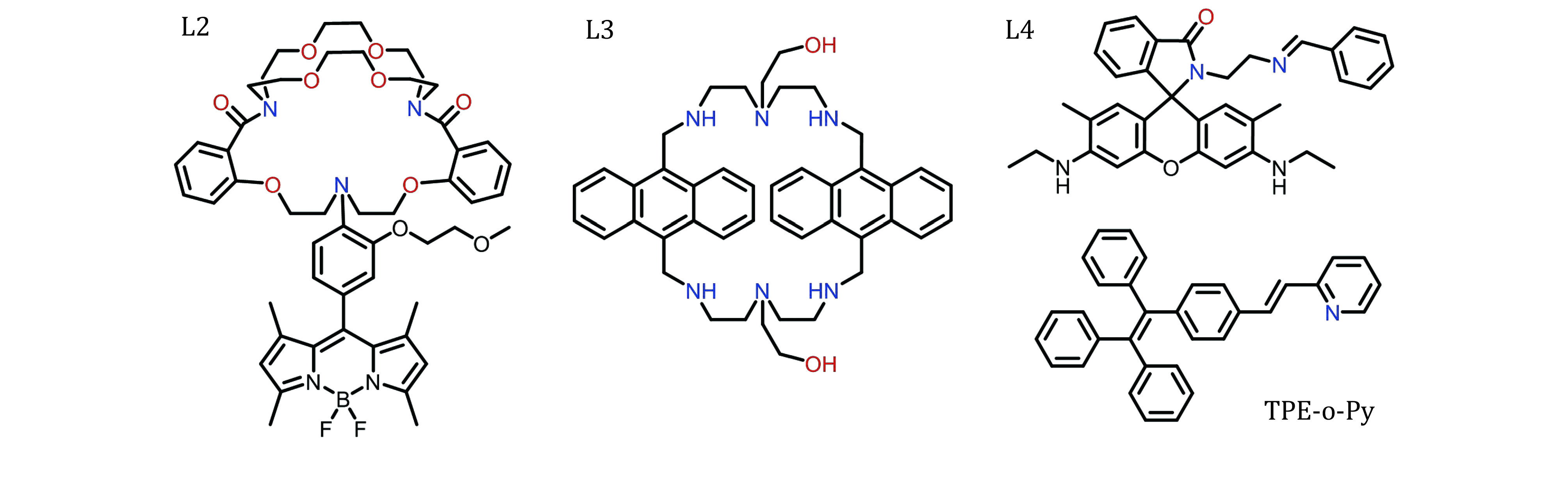
Selected structures of Fe^3+^ probes. Turn-on sensors. Chelation: L2 and L3; Spirolactam ring-opening: L4 and ratiometric probe TPE-o-Py

In contrast to the growing usage of chemical probes for bioimaging of Fe^2+^, measurement of Fe^3+^ in biological environments is rarer. Sahoo *et al*. reviewed advances on Fe^3+^-specific fluorescent probes (Sahoo and Crisponi 2019; Sahoo *et al*. 2012). Due to the paramagnetic nature of ferric iron, its previous recognition by fluorescent probes involves different quenching mechanisms, which is improper for bioimaging applications. A new class of turn-on Fe^3+^ sensors can be classified into two major categories, chelation and spirolactam ring-opening (Fig. 13). Furthermore, some ratiometric sensors recognizing the metal with an appropriate ligand group have been proposed to interrogate intracellular Fe^3+^ ions.

(A) Turn-on Fe^3+^ probes

(a) Chelation

The modularity of the PET platform has been applied to developing Fe^3+^-triggered fluorescent turn-on probes with enhanced properties. A novel PET-based reversible fluorescence turn-on probe L2 for selective assay of Fe^3+^ was introduced (Sui *et al*. 2014). The probe was comprised of BODIPY fluorophore with a 1,10-diaza-18-crown-6-based cryptand that acted as the analyte binding unit. Upon addition of Fe^3+^ in aqueous media (H_2_O/CH_3_CN = 9:1), the weakly fluorescent L2 (PET process is active) displayed 23-fold fluorescence enhancement at 512 nm (*λ*_ex_ = 480 nm). The significant fluorescence enhancement occurred due to inhibition of PET from cryptand to BODIPY fluorophore upon complexation with Fe^3+^. The probe displayed a LOD of 0.13 μmol/L and was applied to sensing intracellular Fe^3+^ ions in HCT-116 cells.

Our group introduced a simple anthracene-based fluorescent turn-on probe L3 containing N^2^-hydroxyethyldiethylenetriamine (HEDTA) for inferring Fe^3+^ concentrations (Qiu *et al*. 2014). When excited at 373 nm, the [2+2] macrocycle fluorescent sensor (20 mmol/L Tris-HCl/MeOH = 1:1, pH 7.2) exhibits weak emission at 398, 421, and 447 nm. In the presence of Fe^3+^, the formation of a L3-Fe^3+^ complex in 1:2 ratio restricts the PET process, causing significant fluorescence enhancement. Probe L3 shows a linear response ranging from 1 to 10 µmol/L with the LOD of 0.58 µmol/L Fe^3+^. Confocal imaging disclosed that the probe possesses the ability of tracing cytosolic Fe^3+^ in SKOV-3 cells.

(b) Spirolactam ring-opening

**Figure 14:**
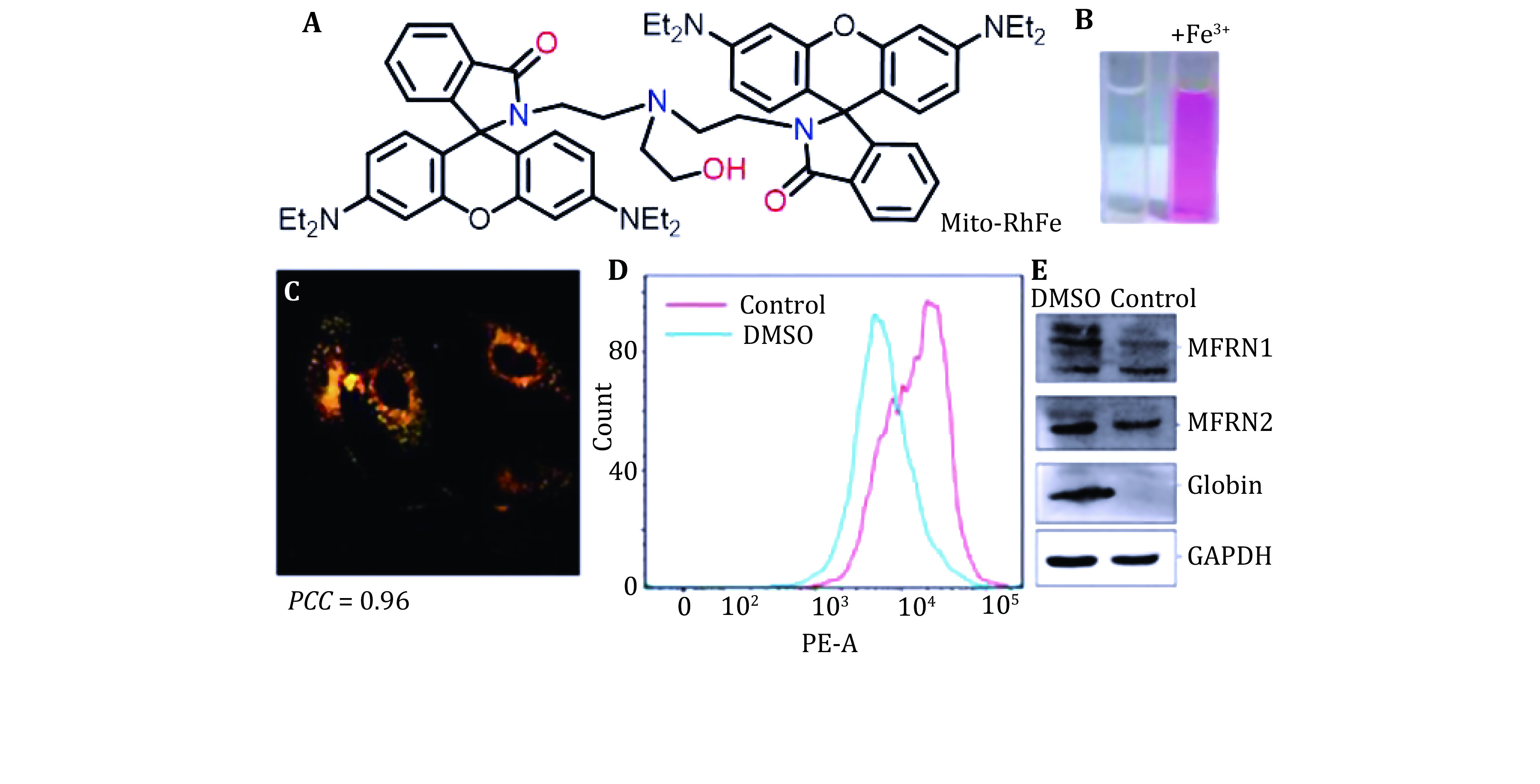
**A** Chemical structure of probe Mito-RhFe. **B** Photograph of Mito-RhFe solution before (left) and after (right) Fe^3+^ addition (20 mmol/L Tris-HCl/MeOH = 1:1, pH 7.2). **C** Colocalization of Mito-RhFe with MitoTracker deep red 633 in MCF-7 cells. The cells were stained firstly by 10 μmol/L probe in PBS for 30 min at 37 °C. **D** Labile Fe^3+^ flow cytometric assay of K562 cells incubated in RPMI1640 medium containing 2% DMSO. **E** Western blot analysis of the cells in **D**. The cells were cultured at 37 °C for 4 days, and stained with Mito-RhFe at 0 °C for 30 min before assay. Cells cultured without DMSO were adopted as the control. Adapted from Zhu *et al*. 2018

Subsequently, we took advantage of the HEDTA ligand to develop another turn-on Fe^3+^ probe Mito-RhFe by appending the HEDTA receptor onto rhodamine B as a cationic, lipophilic tag to localize the probe to mitochondria (Zhu *et al*. 2018). In UV−Vis Fe^3+^ titration of this probe (20 mmol/L Tris-HCl/MeOH = 1:1, pH 7.2), the solution color changes from colorless to magenta, thereby offering Mito-RhFe as an Fe^3+^-induced ‘naked-eye’ chemosensor. With this probe, the mitochondrial labile Fe^3+^ fluctuation in adherent HeLa cells (*PCC* = 0.90) upon ferric ammonium citrate (FAC) incubation was visualized via confocal imaging, and the flow cytometric assay for mitochondrial Fe^3+^ in suspension MEL cells, which is difficult to be monitored in an imaging manner, was also developed. Erythroid differentiation is normally accompanied by hemoglobin biosynthesis and upregulation of the mitochondrial iron importing protein mitoferrin-1 (MFRN1) containing ISC (Shaw *et al*. 2006). Interestingly, the labile Fe^3+^ drop in mitochondria of human K562 erythroleukemia cells undergoing the DMSO-stimulated erythroid differentiation was observed for the first time (Fig. 14).

In addition to Zn^2+^ and Cu^2+^, Fe^3+^ can trigger the hydrolysis and spirolactam ring-opening process of rhodamine derivatives, which has provided a valuable method to design Fe^3+^-selective luminescent sensors with a turn-on response. Lee *et al*. have presented six rhodamine-derived Schiff base probes' selectivity of the Fe^3+^ ion in biological systems (Lee *et al*. 2016). After exposure to Fe^3+^, the probes appeared a new absorption peak at 526 nm, with a concomitant emission maximum at 551 nm. Confocal microscopic analysis showcased that these probes underwent turn-on changes caused by cellular Fe^3+^ ions, and demonstrated a largely enhanced fluorescence upon iron overloading of the HepG2 cell line. They also found that the Fe^3+^-mediated fluorescence augmentation was mainly localized in the ER. Among them, probe L5 exhibited the highest selectivity for ER localization (*PCC* = 0.7854) over other organelles.

(B) Ratiometric sensors for Fe^3+^

Ratiometric probes are highly valued because their intrinsic internal standard can correct potential variations in light intensity, dye localization, and other experimental imaging conditions (Ackerman *et al*. 2017). The aforementioned two turn-on platforms can be adapted to quantify labile Fe^3+^ concentrations in a ratiometric readout, such as a bithiazole derivative (Geng *et al*. 2016), and a spirolactam analog RQBTE (Das *et al*. 2016)

**Figure 15:**
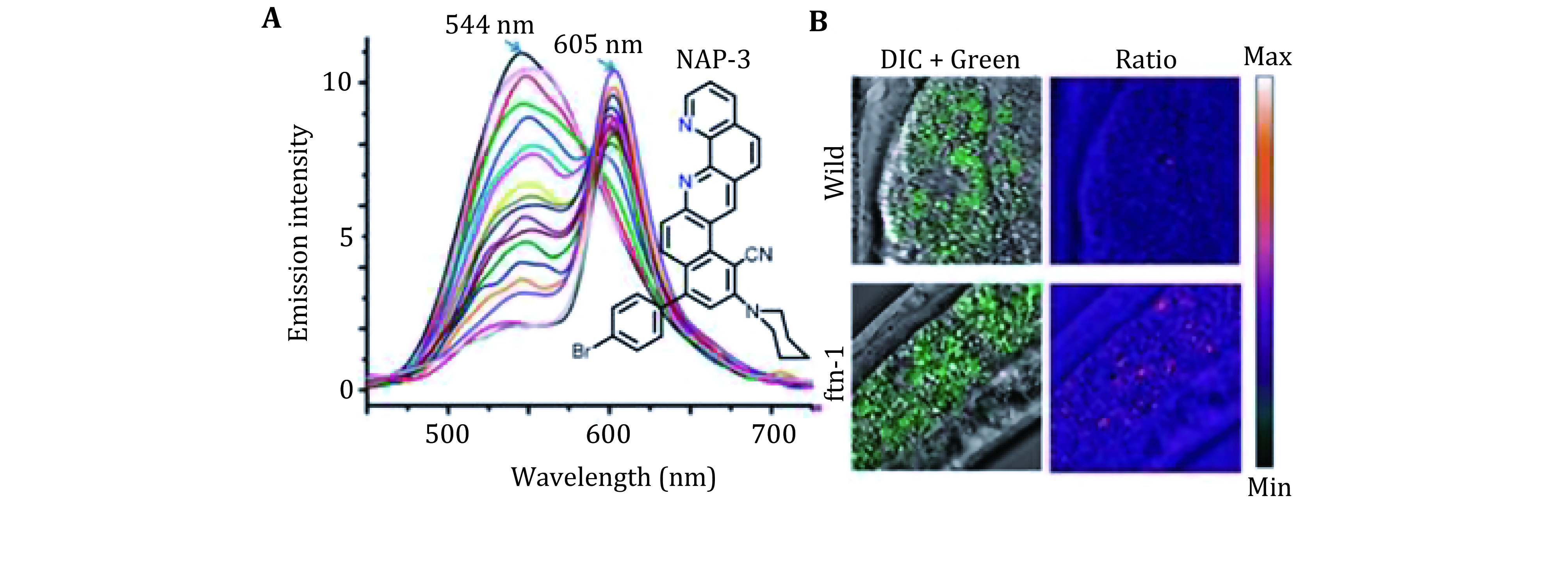
**A** Ratiometric fluorescence spectra (DMSO/H_2_O = 1:9, pH 7.4) of 25 μmol/L NAP-3 (*λ*_ex_ = 365 nm) after 1 h upon addition of increasing concentrations of Fe^3+^ (0–0.68 mmol/L). Inset in **A**: chemical structure of NAP-3. **B** Wild-type and ftn-1 silenced *C. elegans* treated with NAP-3 (3 μmol/L, 24 h). Green channel images obtained with a bandpath of 505–550 nm upon excitation at 405 nm, red channel images obtained with 575 nm long pass upon excitation at 561 nm, and the corresponding ratiometric images (F_red_/F_green_). Adapted from Goel *et al*. 2015

It has been reported that ftn-1 (encoding the iron sequestering protein FTN) is upregulated in the presence of exogenous FAC supplementation, and downregulated upon treatment with an iron chelator deferoxamine (DFO) in the nematode *Caenorhabditis* (*C*.) *elegans* (Valentini *et al*. 2012). Goel *et al*. created the first dual colorimetric and ratiometric fluorescent Fe^3+^ probe NAP-3 (Goel *et al*. 2015), consisting of a naphtho[2,1-*b*]-[1,10]-phenanthroline (NAP). After exposure to increasing concentrations of Fe^3+^ ions (DMSO/H_2_O = 1:9, pH 7.4), a new peak with maximum at 605 nm emerged and the peak at 544 nm decreased in the emission spectrum, with a final enhancement factor (I_605_/I_544_) over 8.5-fold. It can approximately measure as low as 9.1 nmol/L Fe^3+^. Possibly due to steric hindrance, Fe^3+^ chelator DFO failed to remove Fe^3+^ from the complex (NAP-3/Fe^3+^). Common ions do not imply any significant interference in the determination of Fe^3+^ ion. The indicator enabled direct visualization of exogenous Fe^3+^ in HepG2 cells, and endogenous Fe^3+^ in a multicellular organism, ftn-1 silenced *C. elegans* (Fig. 15).

In fact, most conventional organic dyes exhibit aggregation-caused quenching (ACQ) when aggregated. Aggregation-induced emission (AIE) phenomena, originating from the restriction of intramolecular motions (RIM), is thus a unique turn-on approach for addressing the ACQ issue (Chen *et al*. 2019a). AIE luminogens (AIEgens) tend to behave in a non-emissive manner when well dissolved in some solvents, but emit intense fluorescence when poorly dissolved by means of forming aggregates (Mei *et al*. 2015). AIEgens have emerged as a powerful methodology for the assay of transition metal ions such as Zn^2+^ (Jung *et al*. 2015), Cu^2+^ (Feng *et al*. 2014), and Hg^2+^ (Chen *et al*. 2016).

By making use of the position-dependent substituent effects, an AIE featured Fe^3+^ probe (TPE-o-Py) from ortho-substituted pyridinyl-functionalized tetraphenylethylene is synthesized by Tang’s research group (Feng *et al*. 2018). From weak blue to strong red emission (peak 472 nm to 573 nm) under 365 nm UV irradiation, TPE-o-Py displays high metal selectivity and oxidation-state specificity (THF/H_2_O = 3:7) arising from the position isomer of ortho-substitution. And it has a low acid dissociation constant (p*K*_a_ ~3.27) that is close to that of hydrolyzed Fe^3+^. More importantly, the light-up fluorescent probe TPE-o-Py can be applied to sensing Fe^3+^ in HeLa cells with a pronounced red shift in fluorescence emission.

It should be noted that the reduction potential in the cytosol favors Fe^2+^ over Fe^3+^ (Aron *et al*. 2018), and Fe^3+^ is poorly soluble at neutral pH in aqueous media. On the other hand, the vast majority of Fe^3+^ probes have not been well suited for endogenous investigations due to a lack of high sensitivity. In this context, exploring improved motifs to decode Fe^3+^ roles in biology is of tremendous significance.

## SUMMARY AND PERSPECTIVES

In this review, we summarized endeavors made in molecular imaging for transition metal cations. Specifically, we highlighted synthetic sensors that are utilized to image zinc, copper, and iron in biological systems (Table 2). Great challenges still remain in the field to be confronted with:

**Table 2:** Summary of selected probes for metal detection

Probe	Modality	Readout	Cells	Biological highlights
DA-ZP1-TPP	FLI	F_529_	HeLa, WPE-1, RWPE-2, PC-3	Mobile Zn^2+^ in mitochondria of healthy vs cancerous prostate cells
LysoDPP-C4	FLI	F_515_	HeLa, PC3, DU145	Cancerous prostate nodules via Zn^2+^-induced fluorescence signaling
CNSB	TPI	F_470_/F_565_	MCF-7	Fluctuation and distribution of Cu^+^ under ER stress in MCF-7 cells
CCL-1	BLI	n.d.	PC3M-luc	Cu^+^ deficiency in a murine model of nonalcoholic fatty liver disease
RPS1	PAI	PA_710_	bEnd.3	Cross the blood–brain barrier for Cu^2+^ visualization in mice with Alzheimer’s disease
NRh	UCL	UCL_730_	HeLa	*In vivo* detection of Cu^2+^ in Wilson disease
LCy7	FLI	F_690_	HL-7702	Labile Fe^2+^ in ER stress-mediated drug-induced liver injury
ICL-1	BLI	n.d.	PC3M-luc	Labile Fe^2+^ accumulation in a murine model of *A. baumannii* infection
Mito-RhFe	FLI	F_580_	MCF-7	Labile Fe^3+^ drop in mitochondria of K562 cells undergoing the DMSO-stimulated erythroid differentiation
NAP-3	FLI	F_605_/F_544_	HepG2	Endogenous labile Fe^3+^ pools in ftn-1 silenced *C. elegans*
“n.d.”: not determined. TPI and UCL belong to FLI.				

(1) Expanding metal probes bioavailable. It is the quality of probes, including solubility, sensitivity and selectivity (3S), not the quantity that matters. Deliberate design of metal sensors with improved bioavailability is of paramount importance. In addition, other transition metals like manganese (Mn) also take active part in health and disease (Avila *et al*. 2013). Yet Mn sensors are still on an early stage, and there have been far fewer small-molecule probes prepared for Mn^2+^ detection (Bakthavatsalam *et al*. 2015; Liang and Canary 2010).

(2) Combining super-resolution techniques. Super-resolution techniques, such as stimulated emission depletion (STED), structured illumination microscopy (SIM), and stochastic optical reconstruction microscopy (STORM), provide a valuable starting point to study analytes in nano-dimension (Kozma and Kele 2018). Application of this technique to molecular probes for mapping metals is an important and arduous task.

(3) Designing dual-responsive probes. Using two selective probes simultaneously to monitor multiple species is troublesome, and it is therefore preferable to pursue dual-responsive single probes, operating as a logic gate in response to two elements (Kolanowski *et al*. 2018). In this case, interrelations between multiple elements in biology can be deciphered. For instance, if a probe is able to synchronously measure Cu^+^ and GSH without any interference from other analytes, more straightforward data would be offered with less work in NAFLD mentioned above.

(4) Mining multi-mode imaging. Given that diverse imaging modes can be complementary, our laboratory recently reported a FLI/PAI dual-modality probe HS-CyBz (Chen *et al*. 2019b), realizing ratiometric *in*/*ex vivo* tracking for stimulated H_2_S fluctuations in mice. Integrating several imaging modes into a single metal probe, should be appropriately achieved in the longer term.

To sum up, roles for labile metals in cells and tissues, have been elucidated to a certain extent using these molecular probes. Continued innovations in exploiting chemical reagents, in cooperation with physicists and biologists, presage further opportunities for uncovering how metal cations work in biology.

## Conflict of interest

Jing Gao, Yuncong Chen, Zijian Guo and Weijiang He declare that they have no conflict of interest.
